# The Nutritional Components of Beer and Its Relationship with Neurodegeneration and Alzheimer’s Disease

**DOI:** 10.3390/nu11071558

**Published:** 2019-07-10

**Authors:** Francisco José Sánchez-Muniz, Adrián Macho-González, Alba Garcimartín, Jorge Arturo Santos-López, Juana Benedí, Sara Bastida, María José González-Muñoz

**Affiliations:** 1 Departamento de Nutrición y Ciencia de los Alimentos, Facultad de Farmacia. Universidad Complutense de Madrid, 28040 Madrid, Spain; 2 AFUSAN Research Group. Universidad Complutense de Madrid and Instituto de Investigación Sanitaria from Hospital Clínico San Carlos (IdISSC), 28040 Madrid, Spain; 3 Departamento de Farmacología, Farmacognosia y Botánica, Facultad de Farmacia. Universidad Complutense de Madrid, 28040 Madrid, Spain; 4 Departamento de Ciencias Biomédicas, Unidad Docente de Toxicología, Facultad de Farmacia, Universidad de Alcalá, 28805 Alcalá de Henares, Spain

**Keywords:** beer, non-alcoholic beer, Mediterranean diet, aluminum, silicon, dementia, Alzheimer’s disease, neuroprotection

## Abstract

The prevalence of degenerative diseases has risen in western countries. Growing evidence suggests that demenia and other cognition affectations are associated with ambient factors including specific nutrients, food ingredients or specific dietary patterns. Mediterranean diet adherence has been associated with various health benefits and decreased risk of many diseases, including neurodegenerative disorders. Beer, as part of this protective diet, contains compounds such as silicon and hops that could play a major role in preventing brain disorders. In this review, different topics regarding Mediterranean diet, beer and the consumption of their main compounds and their relation to neurological health have been addressed. Taking into account published results from our group and other studies, the hypothesis linking aluminum intoxication with dementia and/or Alzheimer’s disease and the potential role of regular beer has also been considered. Beer, in spite of its alcohol content, may have some health benefits; nonetheless, its consumption is not adequate for all subjects. Thus, this review analyzed some promising results of non-alcoholic beer on several mechanisms engaged in neurodegeneration such as inflammation, oxidation, and cholinesterase activity, and their contribution to the behavioral modifications induced by aluminum intoxication. The review ends by giving conclusions and suggesting future topics of research related to moderate beer consumption and/or the consumption of its major compounds as a potential instrument for protecting against neurodegenerative disease progression and the need to develop nutrigenetic and nutrigenomic studies in aged people and animal models.

## 1. Introduction

Increased lifespan in western countries has resulted in an impressively increased frequency of neurodegenerative diseases, the most common one being Alzheimer’s disease [1]. According to WHO, it has been estimated that people with dementia in the world will reach 74.7 million in 2030 and 131.5 million in 2050 [2]. 

Neurovascular dysfunction, inflammatory processes, oxidative stress and mitochondrial dysfunction are critical factors in the pathogenesis and development of neurodegenerative diseases [3]. Alzheimer’s disease has been pathologically characterized by neuronal degeneration, amyloidal plaques deposition, and neurofibrillary tangles in the brain of affected patients. The decrease in autophagy and the capacity to regulate brain-derived neurotrophic factor (BDNF) production have been reported, among others, as disease-emergent factors [4,5]. The neurodegeneration and hippocampal atrophy are even present at early phases of the disease [6].

While age and apolipoprotein E (APO-E) status represent nonmodifiable risk factors for this disease, diabetes, midlife hypertension, obesity, depression, smoking habits, cognitive inactivity, and low education are some of the known potentially modifiable factors [7]. Recently, the link between obesity and/or the metabolic syndrome with neurodegenerative diseases was proposed [8,9]. Along this line, Nuzzo et al. [10,11] evaluated how obesity and Alzheimer’s disease markers are associated with inflammation, adipokine dyshomeostasis, oxidative stress, mitochondrial dysfunction, and neurodegeneration improvement in the frame of an insulin resistance model or in a functional ingredient-enriched high fat diet intervention,. Further, other factors such as chronodisruption [12], autophagy capacity [13], or mitochondriogenesis [14] have been proposed to influence cognitive disorders. In addition, some pathogens such as the *Treponema* and *Borrela burgdorferi*, and some viruses such as the type I herpes simplex (HSV-1), have been suggested to provoke chronical infections that have narrow links with the etiology of Alzheimer’s disease. It has been reported that amyloid beta peptide (Aβ) acts as an antimicrobial; thus, its accumulation would amplify the immune-response and the subsequent brain inflammation [15]. In addition, epigenetic factors, such as the exposition to some toxics, like lead and aluminum [16,17,18], have been proposed as a risk factor of dementia. It is well known that lead is a neuronal enhancer of oxidative stress, as it induces free radical accumulation and is able to modify the cellular patron of genome methylation [16].

Alzheimer’s disease is characterized by a recurrent situation of cortical neuronal atrophy and destruction that mainly affect the parietal and temporal lobules, compromising three fundamental processes for the integral neuronal maintaining: (a) synapsis or interneuronal communication; (b) metabolism; and (c) reparation. The interruption of any of these processes means a cellular dysfunction that can culminate in apoptosis. Neuronal death provokes memory failure, personality changes, and other manifestations. The histopathological signs of Alzheimer’s disease are neuritic plaques (composed of extracellular deposits of the Aβ protein) and neurofibrillary tangles (mainly composed of the tau protein) [19]. 

Several factors such as statins, light-to-moderate alcohol-consumption, Mediterranean diet-adherence, high educational attainment, physically and cognitively stimulating activities, and APO-E metabolism appear associated with a decreased risk of Alzheimer’s disease onset. On the other hand, while APO-E remains the strongest predictor of Alzheimer’s disease, aging, diabetes mellitus, smoking habits, and lower socioeconomical engagement, among others, are associated with increased risk of Alzheimer’s development [20,21].

## 2. Aluminum as an Alzheimer’s Disease Risk Factor 

Aluminum is the most abundant metallic element in the Earth’s crust and can be found in measurable quantities in food, soil, water, and air. The presence of aluminum in water contributes highly to daily aluminum consumption. Although this metal is normally found in trace amounts in the drinking water, the possibility for low-dose chronic exposure should not be discarded [22]. In addition, the widespread use of products made from, or containing, aluminum ensures the presence of this metal in our bodies. 

Several years have passed since the first time that experts strongly claimed that human exposure to aluminum should be limited as it can exert deleterious effects even at small concentrations [23,24]. Nonetheless, it should be pointed out that the deleterious effects of aluminum in healthy individuals, though inevitable, will be low at normal exposure levels because of their low gastrointestinal uptake and bioavailability, and relatively high urinary excretion [25].

The hypothesis linking aluminum consumption and Alzheimer’s disease, although highly controversial, has been supported by several epidemiological studies [26,27]. In addition, several studies in animal models have given light to this relationship. Therefore, experimental studies in rats and mice showed that aluminum accumulates in the brain cortex, hippocampus, and cerebellum [28], promoting the phosphorylation and aggregation of highly phosphorylated proteins, such as tau protein [29]. Other authors [17] have reported that the amygdala and the hippocampus are the brain areas with the highest aluminum content in an Alzheimer’s disease model. In addition, Oshiro et al. [30] reported that aluminum accumulates more in glial cells than in neurons.

The brain has been found to be the target organ for aluminum accumulation; hence, this element can be primarily considered as a neurotoxic [31]. According to Kawahara [32], this metal induces in vivo as well as in vitro neuronal apoptosis. Aluminum may play an active role in the pathogenesis of critical neuropathologic lesions in Alzheimer’s disease and other related disorders, through cross-linking hyperphosphorylated proteins [33,34]. In fact, Al-induced Alzheimer-like pathological changes were first attributed to tau proteins. Nevertheless, several biochemical, toxicological, cellular, and genetic studies have supported the “amyloid cascade hypothesis”, which explains that the accumulation of Aβ protein (AP) and its neurotoxicity play a central role in the pathogenesis of Alzheimer’s disease [35]. 

Walton [36] and Bolognin et al. [37] suggested that aluminum is engaged in the brain’s neurofibrillary tangles formation by promoting the expression of the Amyloid precursor protein (APP) of the AP and increasing the levels of β-40 and β-42 fragments in the brain and should, therefore, be considered as a causative factor in Alzheimer’s disease. In addition, aluminum appears to be associated with AP in the brain [38,39], as the chronic application of this metal caused the accumulation of AP in cultured neurons of rat cerebral cortex and in neuroblastoma cells. It is known that the monomeric form of AP has a random coiled structure, while the oligomeric AP have pleated sheet structures and form insoluble aggregates, named amyloid fibrils. The neurotoxicity of AP peptides has been studied in an aging model compared to freshly prepared AP in cultured neurons, and it has been demonstrated that the soluble oligomers are synaptotoxic and neurotoxic [35]. On the other hand, this metal can cause pro-oxidant activity. Exley [38] reported that this effect might be explained by the formation of an aluminum superoxide semireduced ion radical (AlO_2_^2+^).

As is known, reactive oxygen species (ROS) interact with all biological macromolecules, including lipids, proteins, nucleic acids, and carbohydrates, contributing to neuronal death and, in turn, to the neuropathology associated with several diseases [28]. Although the exact mechanism by which the metal may influence disease processes remains unknown, an increase in oxidative stress and inflammatory events, two major causes of neurological diseases, have been proposed. Aluminum initiates and propagates an inflammatory response within the aging brain, suggesting that this may be one of the mechanisms by which the metals induce neurodegeneration [22]. In Alzheimer’s disease transgenic mice models, dietary aluminum markedly increased lipid peroxidation and Aß-level presence [40]. In isolated systems, aluminum may increase the oxidative stress produced by transition metals such as iron [41] or copper [42]. In line with this observation, our group recently reported that aluminum intoxication contributes to a metal imbalance in the brain, which in turn would be responsible for this organ oxidation and reduced antioxidant capacity [43]. Our results are in line with those of several authors who described that Al3+ decreased the activity of the antioxidant enzymes catalase (CAT), superoxide dismutase (SOD), and glutathione peroxidase (GPx) [44,45]. Sharma et al. [46] found an increase of oxidative stress in the brain and serum with low reduced glutathione (GSH), GPx, CAT, and SOD levels after 10 weeks of aluminum chloride gavages exposure. Moumen et al. [47] reported increased concentrations of tiobarbituric acid reactive substances (TBARS) and glutathione S-transferase after aluminum intoxication. 

Other studies have demonstrated that aluminum intoxication increased brain TBARS levels and tumor necrosis factor alpha (TNFα) expression, suggesting that oxidative stress and neuroinflammation was induced [48]. The induction of these processes has been proposed to be pathogenic for early events of Alzheimer’s disease. Thus, brain TNFα-rise has been shown to precede development of the disease in patients with mild cognitive impairment [49]. In line with the hypothesis that aluminum plays an active role in neurodegenerative diseases, Campbell [22] and Becaria et al. [50] found that brain TNFα expression was increased in mouse brains exposed to aluminum when compared to the control group. According to Lukiw et al. [51] aluminum exposure induced inflammatory gene expressions in primary neural cells.

Furthermore, aluminum exposure has been associated with the impairment of the cholinergic system by altering cholinergic projection function and structure, suggesting how this metal could contribute to the pathological process in neurodegenerative progression [52]. Martinez et al. [53] concluded that aluminum increased hippocampal reactive oxygen species and lipid peroxidation, reduced antioxidant capacity, and decreased acethylcholinesterase (AChE) activity.

It has been described that in Alzheimer’s disease, AChE expression is substantially altered, and its activity is decreased in most brain regions. However, AChE activity is increased within and around the Aβ plaques. Noremberg et al. [18] showed the contrary, that is, a decrease of the AChE activity in the presence of aluminum in the hippocampus and cortex, which would be a precursor of Alzheimer’s disease. It is important to emphasize that since cerebral AChE is an important regulator of behavioral process, the decreased AChE activity found in the cortex and hippocampus may be an indicator of aluminum-induced damage in the brain. Kaizer et al. reported a decrease of AChE activity in the hypothalamus but verified an enhancement in the striatum area and no alterations in the hippocampus, cortex, and cerebellum [54]. These results demonstrate that aluminum acts differently depending on the dose and chemical form of Al^3+^ administration, the administration route (oral or intraperitoneal), and the time of exposure. Therefore, aluminum could produce a dose-dependent effect on AChE, stimulating AChE at low levels or short exposures and inhibiting AChE at high doses and/or long exposures periods. This polarized effect of aluminum on the AChE activity may be due to the direct effect of the metal or due to the peroxidation-induced changes in the structure of membrane following aluminum exposure [55].

**Figure 1:**
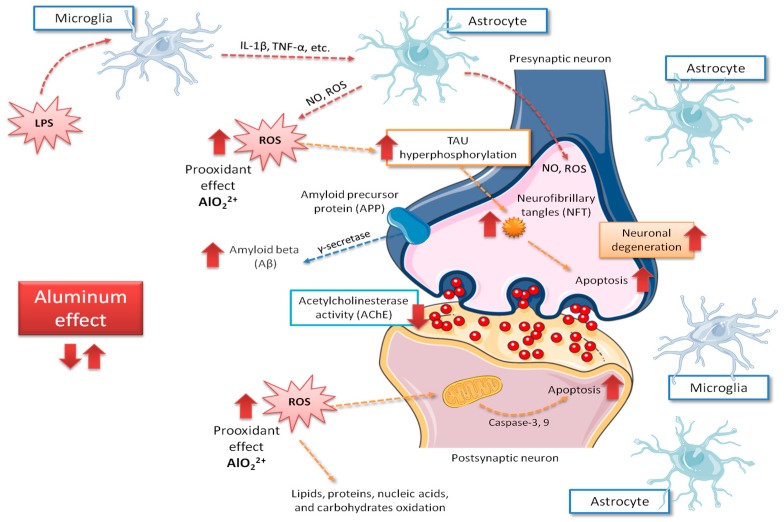
Main central aspects related to neurological mechanisms implicated in Alzheimer’s disease caused by aluminum intoxication. Red arrows indicate the increase of the possible negative effects of aluminum exposure on the brain, those including ROS production increase and, in consequence, rise on tau phosphorylation, apoptosis, amyloid beta peptide (Aβ) accumulation and neurodegeneration. IL-1β, interleukin 1-beta; LPS, lipopolysaccharide; NO, nitric oxide; ROS, reactive oxygen species; TNFα, tumor necrosis factor alpha.

Figure 1 shows a summary of the main deleterious effects of aluminum on brain cells. Although Oshiro et al. [30] reported that aluminum is highly accumulated on glial cells, the importance of those findings has been poorly discussed, and most studies have been centered on neurons’ function. 

## 3. Nutritional Factors and Neurological Health—Beer as a Component of the Mediterranean Diet

Cognition at various levels has been consistently associated with the nutritional status that in turn depends on the intake of specific nutrients or food ingredients [56], specific foods [57] or particular dietary patterns [58]. Among the most known factors, diet quality has been related to the hippocampus volume (the brain structure that is mainly associated with learning and memory) [59]. In elderly humans, the Mediterranean diet has been associated with reduced atrophy of the brain [60] and reduced amyloid peptides load [61]. In addition, the continuous stress induces the cortisol hormone to increase, a fact that can be a link between chronodisruption and the neurodegenerative disease as a consequence of distortion at neuronal renovation by cortisol at the paracortical gyrus [62,63].

Based on large scientific evidence, the Mediterranean diet has been considered to be one of the healthiest diets [64,65,66] due to its high nutritional quality. In fact, when the level of adhesion to a Mediterranean diet model is optimal, there is a reduced risk of inadequate nutrient and bioactive compound intakes, or it has even been positively related with an increase in longevity [67,68,69]. The characteristics of this diet have been frequently summarized as a pyramid in which the frequency of food consumption is highlighted. Since their creation by Keys et al. [70], several modifications to the original pyramid have been proposed, trying to adapt the original one to present time or even considering the Mediterranean diet as an integral temple of life [71]. Thus, tridimensional pyramids [72], tables, and new figures [73] have appeared, showing some similarities to the original one, increasing the presence of new dietary compounds and dishes, and supplementary lifestyle information. It is well known Mediterranean diet is characterized by a high proportion of food of vegetable origin, where the presence of virgin olive oil is mandatory [74,75]. Fish, cheese, and yogurt are moderately consumed, while meat is rarely consumed alone and always forms part of complex dishes. Wine or beer during main meals is also one of its characteristics [76]. It is well known that the Mediterranean diet has been recognized to be an intangible heritage of humanity and due to its composition plurality, several authors [77,78] justify the use of the term Mediterranean diets instead of Mediterranean diet. 

Beer is one of the most consumed alcoholic beverages around the world. Table 1 shows data on beer consumption of most representative countries belonging to the different continents. The Czech Republic shows the highest per head consumption, with more than 150 L per year. There are other relevant beer consumers with more than 100 L/head/year. China is the highest consumer in the world, although its per habitant consumption is under 30 L/year [79].

There is growing evidence from large-scale, population-based studies that long-term adherence to the MD may help to protect against dementia and preserve brain and cognitive function in the later stage of the lifespan [80,81,82,83,84]; however, negative–nonpositive information on the Mediterranean diet on this topic is also available [84,85,86].

According to the PREDIMED study, moderate beer-drinkers have a healthier lifestyle and display an overall dietary pattern closer to that of people following the traditional Mediterranean diet than their nondrinker counterparts (more cereals, legumes, vegetables, fish, and olive oil and fewer dairy products), although they also show a greater consumption of meat and meat products [87]. These authors also reported fewer cardiovascular disease risk factors among beer-drinkers that partially explain the protective role of beer in the development of atherosclerosis and cerebrovascular diseases. 

**Figure 2:**
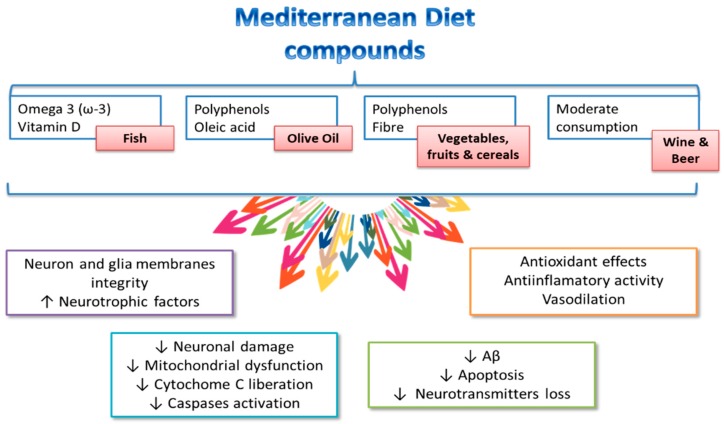
Components of the Mediterranean diet and potential mechanisms influencing cognitive health. Modified from El-Swefy and Atteia [20].

Figure 2 shows some of the Mediterranean diet components from which a positive influence on cognitive health has been described. It has to be pointed out that the effect of the Mediterranean diet is due to its particular foodstuff component, and thus, it appears to be linked to its whole nutrient profile [76,88,89], partially explaining the positive effects of a high Mediterranean diet adherence on brain health [90,91,92,93,94].

**Figure 3:**
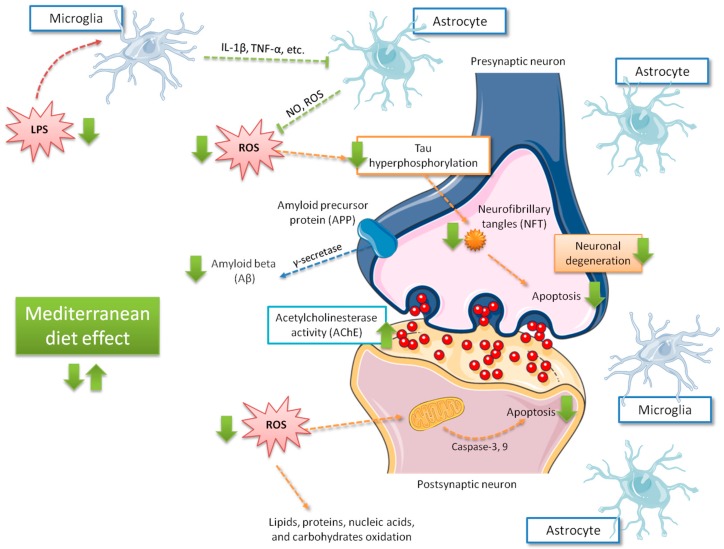
Main effects related to Mediterranean diet adherence on cognitive function. Green arrows indicate the increase of the possible effects on brain, those including ROS production decrease and, in consequence, less tau phosphorylation, apoptosis, Aβ accumulation, and neurodegeneration. IL-1β, interleukin 1-beta; LPS, lipopolysaccharide; NO, nitric oxide; ROS, reactive oxygen species; TNFα, tumor necrosis factor alpha.

Some mechanisms have been proposed to explain the beneficial effects of the Mediterranean diet on mild cognitive impairment [20,95,96] (summarized in Figure 3). Gardener et al. [96] found that Mediterranean diet adherence was associated with a reduced number of strokes and lower incidence in Alzheimer’s disease and mild cognitive impairment development in elderly individuals. In addition, the Mediterranean diet may confer its favorable effects in cognitive function due to its antioxidant [97] and anti-inflammatory properties [98]. Oxidative stress has been largely associated with cognitive decline and neurodegenerative disorders [99], while inflammation has been linked to vascular health impairment, as well as brain damage, through amyloid peptide accumulation and subsequent activation of astrocytes and microglia [100]. Abuznait et al. [101] proposed that the Mediterranean diet induces an increase of neurotrophic factors related to neurotransmission, synaptic plasticity, and elimination of Aβ from the brain. The positive Mediterranean diet effects on the pathogenesis of vascular disease and Alzheimer’s disease have largely been linked to the reduction of oxidative stress, through the consumption of abundant antioxidant and anti-inflammatory agents (e.g., polyphenols) which, in turn, may alter the expression of inflammatory markers. Thus, supplemental foods such as extra virgin olive oil and nuts, particularly rich in phenolic compounds [102], may counteract oxidative processes in the brain by reducing, in turn, neurodegeneration. Other additional mechanisms attributed to polyphenols, such as cerebrovascular blood flow improvement, BDNF synthesis enhancement, neuronal signaling modulation, and neurogenesis stimulation should ameliorate neurologic health [103]. Nonetheless, the presence of other foods (such as fish rich in omega-3 polyunsaturated fatty acids) regularly found in the Mediterranean diet are also implicated in the reduced risk of cognitive decline and dementia of people showing high adherence to this diet. Light to moderate alcohol use may be associated with a reduced risk of incident dementia and Alzheimer’s disease [104].

## 4. Composition of Beer—Beneficial Aspects on Alzheimer’s Disease

Beer, a beverage probably originating in Mesopotamia with the Assyrians, Sumerians, and Babylonians (XXIV century B.C.), is one of the oldest recorded recipes. The brewing process was first documented on papyrus scrolls by ancient Egyptians [105]. Later, and due to barley crops being abundant, the brewing process extended to North Europe, being a safe alternative to drinking water. Monks were very much the foremost brewers of the Middle Age, with virtually every monastery having one brewery on site [106].

As is known, beer can be classified according to bottom or top fermentation yeast. Top-fermented beers include brown ale, mild ale, old ale, pale ale, stout, and wheat beer. The most commonly consumed types of beer in the world are pale lagers, which normally use a bottom-fermenting yeast. Main lagers include pale lager, bock, dunkel, helles, oktoberfestbier/märzen, pilsner, schwarzbier, and Vienna lager. In addition, there are non-alcoholic beers aimed at sectors of the population that do not want or cannot drink alcohol. The consumption of beer has grown during the last few decades, mostly among young adults. Currently, it is one of the most consumed beverages in the world, as shown in Table 1.

Composition can be different from one beer type to another; however, the average beer contains a not insignificant amount of nutrients, such as carbohydrates, protein/amino acids, minerals, vitamins, and other compounds, such as polyphenols (Table 2 and Table 3) [76,88]. Among minerals, potassium, phosphorus, calcium, sodium, and silicon are the most abundant, while folic acid is the most abundant vitamin, which has even pushed nutritionists to consider this drink as a valuable source of folic acid, as a can of beer (330 mL) contains 20–25 µg folate, an amount that covers 10%–15% and 5%–7% of the recommended intakes for this vitamin in men and women, respectively [107] (Table 2). In addition, beer has been considered a relevant source of some bioactive compounds with physiological properties (Table 3).

**Figure 4:**
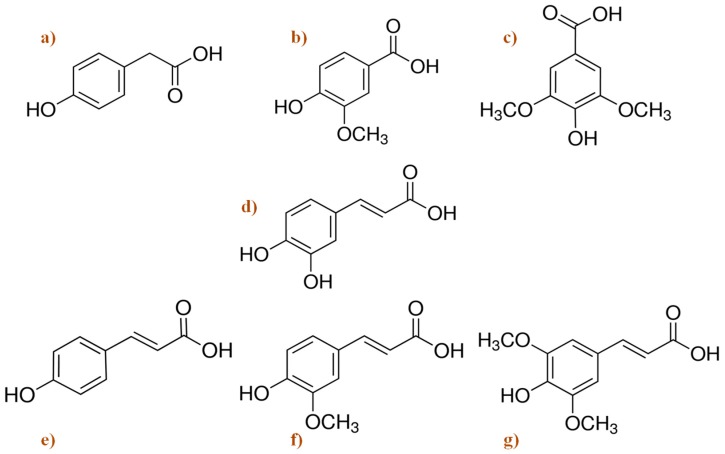
Chemical structure of the main phenolic acids found in beer. (**a**) 4-hydroxyphenylacetic acid; (**b**) vanillic acid; (**c**) syringic acid; (**d**) caffeic acid; (**e**) p-coumaric acid; (**f**) ferulic acid; and (**g**) sinapic acid [109].

Figure 4 shows the general chemical structure of most representative compounds found in beer. Among several components, beer contains the phenolic acids 4-hydroxyphenylacetic, vanillic, caffeic, syringic, p-coumaric, ferulic, and synaptic acids. Alkaline hydrolysis experiments show that most of the phenolic acids are present as bound forms, and only a small portion can be detected as free compounds [108]. 

Beer can also be an alcohol source, although its content is rather variable (0%–15% Vol.) depending on the type, ingredients, and fermentation modality. Most available regular types of beer contain 4%–5% alcohol volumes equivalent to 3.2–4 g alcohol/100 g or 100 mL. A non-alcoholic beer (also called beer without, beer low in alcohol or loose beer) is a beer with a very low or no alcohol content. Most non-alcoholic beers are lager, but there are also some ale varieties. There are four types of non-alcoholic beers: alcohol-free, dealcoholized, low-alcohol, and alcoholic beer. In the European Union, beer cannot contain more than 1% alcohol by volume to be labeled as “alcohol-free”. In the UK, the legislation stipulates that beer can be labeled as non-alcohol or alcohol-free (‘non-alcoholic’) when its content does not exceed 0.05% by volume, as dealcoholized up to 0.5% and low-alcohol (‘low in alcohol’) up to 1.2%. In the United States of America, beverages containing less than 0.5% alcohol by volume are considered non-alcoholic [109,110]. Every day, more and more non-alcoholic or low-in alcohol-beers are available and appreciated. Spain appears as the consumers’ leader of non-alcoholic beer in Europe, with a 14% rate of total beer consumption, almost triple that of its neighbor, France (INSERM) [110].

Several studies in both animals and humans on the potential brain’s health benefits of regular beer consumption and of main representative compounds of beer have been realized and are referenced and detailed in Table 4. 

Thus, the Helsinki Sudden Death Autopsy Series study, carried out in 125 males, concluded that beer consumption might protect against Aβ aggregation in the brain [111]. The alcohol contained in the beer, apparently, can also exert a neuroprotective effect. This protection appears linked to signal transduction activation processes potentially involving ROS, several key protein kinases, and increased heat shock proteins [112]. In fact, significant reduced risks of cognitive loss or dementia in moderate, nonbinge consumers of wine, beer, and liquor have been observed. Downer et al. [113], using data from the Framingham Heart Study Offspring Cohort, found that alcohol consumption status in late life, but not in midlife, was associated with episodic memory and hippocampal volume. Compared to late life abstainers, moderate consumers had a larger hippocampal volume, while light consumers had higher episodic memory.

Hops and silicon are two of the most important components of beer, and their composition and effects are briefly described in the following subsections.

### 4.1. Hops (Humulus lupulus L.)

Hops, one of the raw materials of beer, brings bitterness and serves as an important source of phenolic compounds. Polyphenols, mainly catechins, flavonoids, phenolic acids, prenylated chalcones, and proantocianidins, comprise about 14.4% of dried hops cones [143]. Around one fourth of polyphenols in beer originates from hops, and the rest belongs to malt [144]. Moreover, hops provide a resin containing monoacyl phloroglucinols that are precursors of bitter acids (e.g., α-acid humulones and iso-α-acids) during beer production. Simple phenols, benzoic acid derivatives and cinnamic acid, coumarins, catechins, di- and tri-oligomeric proanthocyanidins, prenylated chalcones, and α- and iso-α-acids derived from hops are different classes of polyphenols in beer (Table 3 and Figure 4). Hops and most common regular beers contain 8-prenylnaringenin, which is a potent phytoestrogen [145]. Hops also contain myrcene, humulene, xanthohumol, isoxanthohumol, myrcenol, linalool, tannins, and resin, as well as 2-metilbutan-2-ol, which is a component of hops brewing [146]. Arranz et al. [76] reported that these beer compounds show different in vitro biological activities, such as antioxidant, anticarcinogenic, anti-inflammatory, estrogenic, and antiviral. According to Ano et al. [138] the iso-α-acids contained in beer may be useful for the prevention of dementia due to their ability to suppress neuroinflammation and improve cognitive function. These authors demonstrated that the consumption of iso-α-acids, the hops-derived bitter compounds in beer, prevents inflammation and Alzheimer’s disease pathology in a mice model, via the regulation of microglia activation, and therefore prevents the inflammation-related brain disorders [140]. The iso-α-acids, as agonists of peroxisome proliferator-activated receptor gamma (PPAR-γ), increase microglia phagocytosis of Aβ and suppress inflammation in neuronal tissue [147]. Xanthohumol has been defined as a very important compound of hops and beer due to its positive effects as an antioxidant and neuroprotective described in some central papers [148]. In this line, Huang et al. [137] found several metabolic pathways where prenylflavonoid xantuhumol can be engaged as protective in some neurodegenerative diseases, such as Alzheimer’s (e.g. through inhibiting the Aβ accumulation and APP processing, and inducing amelioration of tau hyperphosphorilation via PP2A, GSK3β pathways in N2a/APP cells).

### 4.2. Silicon

Silicon, in the form of silicic acid or orthosilicic acid, is mainly found in whole grains (e.g., cereals) and fiber-rich foods. Therefore, due to its production ingredients, beer is one of the main silicon dietary source [88,119]. 

Although the health benefits of silicon, with regard to skeletal and neurological function and status, have already been recognized [149], there is currently limited available information regarding the possible beneficial effects of silicon on neural toxicity. In this regard, recent papers of our group (Table 4) have clearly demonstrated the antioxidant properties of silicon in neuroblastoma cells and rat’s liver [128,150].

Noremberg et al. [18] showed that intraperitoneal administration of silicon in similar concentrations to those found in parenteral nutrition reduces the harmful effects of increased lipoperoxides (LPO) in rat brain induced by long-term aluminum exposure. This finding is relevant because of oxidative stress, and increased LPO levels in cerebral tissue are major factors in the development of neurodegenerative diseases [151]. The reduction of TBARS levels by the administration of silicon strongly suggests the neuroprotective effect of this metal. Silicon was also effective in reducing the LPO, since the formation of hydroxyaluminosilicates may reduce aluminum availability, leading to a decrease in ROS generation. Therefore, silicon could be considered a protector against aluminum-associated neurological diseases [126].

## 5. Effects of Beer on Aluminum Bioavailability

Silicon and silicic acid may decrease aluminum bioavailability by partially blocking its gastrointestinal tract uptake [152] and by impeding its reabsorption [153]. According to Gillette Guyonnet et al. [127], silica is probably the natural antidote of aluminum and could play a beneficial role by decreasing aluminum bioavailability. These authors suggest the possible use of silicates as a therapeutic agent for Alzheimer’s disease, since both model tangles and precipitated β-pleated sheets of Aβ4 can be reversed to soluble forms by silicates. Likewise, the same authors found that silica in drinking water might reduce the risk of developing Alzheimer’s disease [126].

More than one decade ago, it was demonstrated that beer intake affected the kinetics of aluminum uptake and excretion. A three-day shot-term study in male mice subjected to the conjoint administration of aluminum (450 μg/ml) and two doses of beer, one equivalent to moderate-low consumption in humans (0.5 L/d) and another equivalent to moderate-high consumption in humans (1 L/d), was performed [120]. Following this study, a long-term test was formulated to substantiate the possible protective action of beer against chronic aluminum exposure and accumulation in brain tissue. Results demonstrated that silicic acid and beer affected the kinetics of aluminum uptake and excretion, possibly through an interaction between aluminum and silicon in the digestive tract. Moreover, silicic acid did not only reduce the aluminum gastrointestinal absorption but also increased the aluminum release and excretion from the body. In fact, the aluminum group excreted significantly lower fecal aluminum than the aluminum–beer and aluminum–silicon groups (487.7 ± 70.8 µg/g feces vs. 581.0 ± 92.6 and 665.9 ± 160.4, respectively) [122]. Therefore, it was hypothesized that silicon in the form of silicic acid may lower aluminum bioavailability and hence should be considered an element that may afford protection against aluminum intoxication.

The dietary o-silicic acid supplement was efficient in lowering aluminum brain depots in the aluminum–silicon mice to the same values observed in the basal group. Similarly, although not significantly, the administration of beer tended to decrease the aluminum content in the brain. These results suggest that beer intake did not produce the same increase in brain silicon levels as the dietary silicon supplement did. Nonetheless, based on these results, it must be suggested that silicon administration appears effective in preventing aluminum accumulation in mouse brain, as was previously reported by Granero et al. [121]. 

**Figure 5:**
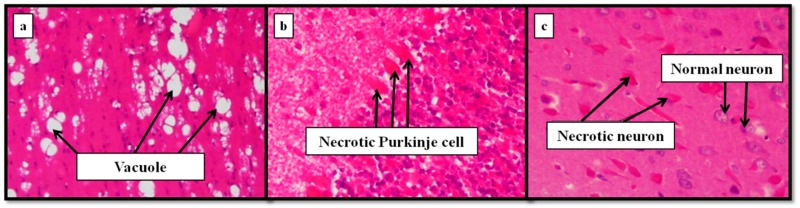
Histological examination of a mouse brain intoxicated with aluminum. (**a**) Spongiosis but no neuronal necrosis in the positive aluminum control mice; (**b**) necrosis in the cortex and in the cerebellum of aluminum-intoxicated animals treated with silicon; (**c**) necrosis of cortical neurons of animals treated with conjoint aluminum and beer [122] (Elsevier copyright license number: 4621240772368).

Figure 5 shows some relevant insights of a mouse brain intoxicated with aluminum [122]. Thus, white matter spongiosis but no neuronal necrosis was observed in the positive aluminum control mice (a). Aluminum-dosed animals treated with silicon showed necrosis both in the cortex and in the cerebellum (b). By contrast, the brains of the mouse that received conjoint administration of aluminum and beer exhibited necrosis of cortical neurons (c). Although the results clearly suggest more advanced lesions in the aluminum-intoxicated mice, it was concluded that more detailed studies of longer duration should be further performed, to validate the pathologic repercussions found.

As already commented on, the effectiveness of silicon could be attributed to its interaction with aluminum through the formation of nontoxic aluminosilicate complexes that decrease free aluminum availability. A number of biological sites have been identified, in which silicon and aluminum are co-deposited or co-localized. Among them, the senile plaque cores in the cerebral cortex of patients suffering from senile dementia/Alzheimer’s type have been more deeply investigated. High-resolution solid-state nuclear magnetic resonance measurements on the central regions of these plaques have shown that silicon and aluminum are present as an aluminosilicate species as a way to partially block aluminum toxicity [154]. Plaque structures have also been observed in mentally normal elderly patients, and the use of dietary silicon supplements as a preventive measure for Alzheimer’s disease has been suggested [155].

## 6. Effect of Beer on Brain Antioxidant and Inflammatory Status

Taking into account the relationships between aluminum exposure, oxidative stress, inflammation, and certain neurological disorders already commented on, our research group also carried out studies to evaluate the neuroprotective effect of beer itself and by means of its major components (silicon and hops) on the oxidative and inflammatory alterations induced by aluminum intoxication in mice. Changes in gene expression of some antioxidant enzymes and inflammatory factors were evaluated in the brains of different mice groups that distinctly received aluminum plus beer, aluminum plus silicon or simply aluminum for 3 months [48]. Results showed that inclusion of silicon in the diet in the form of beer or silicic acid reduces the harmful effects of increased cerebral peroxidation by lowering aluminum levels in the brain. In addition, silicon, silicic acid or beer highly blocked the prooxidant and pro-inflammatory actions of aluminum by decreasing brain TBARS levels and glutathione peroxidase (GPx) and tumor necrosis factor-alpha (TNFα) expressions but increasing the superoxide dismutase (SOD) and catalase (CAT) enzyme expressions. Thus, the changes on redox status induced by beer or silicon consumptions seem to be related with an adequate ROS production, giving rise to a correct reduced–oxidized glutathione (GSH-GSSG) balance. These findings are relevant, as oxidative stress and increased lipid peroxidation in the brain are the major contributing factors for neurodegenerative disease development [17,156,157,158]. The administration of silicic acid or beer reduced TBARS levels, strongly suggesting the neuroprotective properties of silicon. Interestingly, brain gene expressions of Mn-SOD, Cu/Zn-SOD, and CAT were positively correlated with one another, but all of them negatively with GPx gene expression, supporting the hypothesis that ingestion of silicon has beneficial effects against aluminum intoxication [48]. 

The lower TNFα expression in the silicic acid and beer groups with respect to the aluminum group and the control group newly suggests the existence of a detoxification mechanism. These results were suggested in previous studies to be related with a successful chelation of aluminum, followed by its mobilization and excretion from the body as previously discussed [122]. Winkler et al. [117] reported that beer components act as anti-inflammatory agents by reducing the effects mediated by pro-inflammatory cytokine interferon-gamma (IFN-γ), and as antioxidant through reducing ROS formation. In fact, hops, due to their high polyphenol content, have been found to exert anti-inflammatory effects [76]. 

Although results due to silicon administration were very relevant, the role of other compounds present in beer, as hops, some polyphenols, folic acid, melatonin, and alcohol, cannot be ruled out. In fact, hops have been found to be a relevant source of resveratrol that could partially explain the improvement of the antioxidant status in beer-administrated mice [125]. In addition, hops decrease production of TBARS and carbonyl groups in the elderly [159]. Folic acid is responsible, through cystathionine-β synthase, for producing cysteine, a precursor of glutathione that exerts antioxidant properties. Moreover, hyperhomocysteinemia—a condition related to low folic acid bioavailability—increased generation of free radicals [160], and several studies in humans have reported an inverse association between homocysteine and cognitive impairment or dementia [161,162]. 

Another interesting beer compound is melatonin [132]. Maldonado et al. [129] suggested that the melatonin present in beer does contribute to the total antioxidant ability of human serum. Therefore, melatonin can directly act as a free radical scavenging and indirectly stimulating the role of some antioxidant enzymes (e.g., SOD, GPx, GR) which, in turn, will reduce the toxicity of radicals and their associated reactants [163]. In addition, melatonin reduced Aβ-induced oxidative stress and the level of IL6 and IL1-β pro-inflammatory cytokines in in vivo studies [132,133,164].

## 7. Effect of Beer on Metal Homeostasis in the Brain

**Figure 6:**
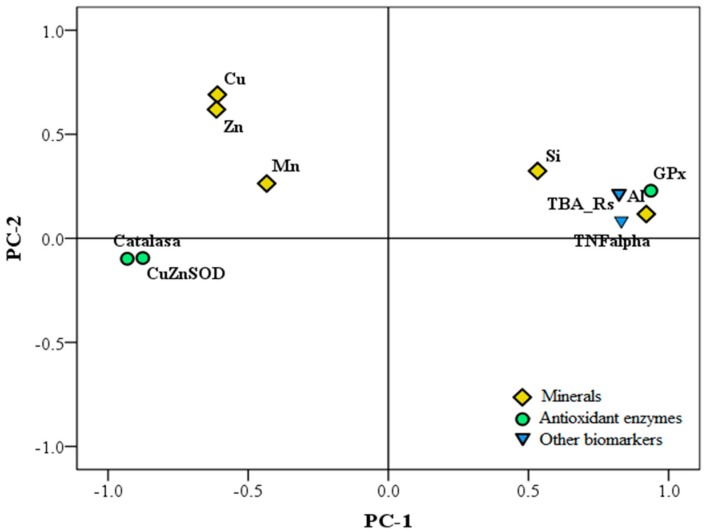
Principal component analysis (PCA) plot of the minerals, oxidation, and inflammation biomarkers of mouse’s brains. The co-administration of aluminum and silicon or beer partially blocked the metal disbalance and reversed the inflammatory and oxidative status in the mouse brain.

Emerging data suggest that aluminum may heighten some events associated with neurodegenerative diseases by inducing mineral imbalance [165]. According to Colomina and Peris-Sampedro [17], the interaction between aluminum and iron modifies iron homeostasis by increasing the intracellular pool of free iron, releasing it from the iron-containing enzymes and proteins, which in turn contributes to a higher ROS production. Maintaining transition metal homeostasis is known to be important in a wide variety of biological functions, such as antioxidant defense mechanisms. Aiming to shed some light on the effect of aluminum on brain metal homeostasis, we evaluated in mice the effect of aluminum exposure on copper, iron, magnesium, manganese, silicon, zinc, and aluminum brain contents and the correlations between those metal levels and some antioxidant status and inflammation markers. In addition, by means of statistical models, we elucidated potential mechanisms that will contribute to explaining the role of the brain metal content on brain toxicity [43]. Aluminum nitrate exposition significantly increased silicon contents in mouse brains but decreased copper, manganese, and zinc levels. Under aluminum nitrate exposition, beer or silicic acid significantly lowered aluminum and silicon levels and normalized those of copper, manganese, and zinc in the brain. The nonsignificant effects found on iron can be partially explained, according to Colomina and Peris-Sampedro [17], based on that aluminum generates labile iron from enzymes and proteins but does not change the total iron content of the brain. A principal component study (PCA) performed considering mouse groups, brain metals, and brain oxidative/inflammatory profiles showed that the aluminum group was clearly separated from control animals, while aluminum–beer and aluminum–silicon were placed closer to control mice, suggesting a partial block of aluminum pro-oxidant effects. On the other hand, pro-oxidant markers in the brain connected with the brain aluminum content and, to a lesser extent, with that of silicon. By contrast, zinc and copper brain levels were closer to the antioxidants’ enzyme activities (Figure 6). Thus, it can be highlighted that the conjoint administration of aluminum nitrate and silicic acid or beer partially blocked the metal disbalance induced by aluminum nitrate and reversed the inflammatory and oxidant/antioxidant status of the mouse’s brain. Such a blocking effect joined to the impact of silicic acid/beer on aluminum nitrate absorption justifies the importance of adjusting dietary silicon levels to aluminum intake.

## 8. Effect of Non-Alcoholic Beer, Silicon, and Hops on Brain Damage and Behavioral Changes Induced by Aluminum

The previous results clearly show the benefits of beer consumption, so it would be appropriate to recommend it as a way to alleviate the deleterious effects of aluminum exposure on the brain. However, regular beer should not be recommended to some population sectors (e.g., pregnant women, metabolic syndrome patients, non-alcoholic fatty liver patients) due to its alcohol content [166]. Therefore, our research group conducted a new study on the capacity of non-alcoholic beer (NA-beer) and its components (silicon and hops) to enhance brain antioxidant and inflammatory status, which in turn would help with improving brain functions, improving the impaired learning ability, and motility caused by aluminum intoxication. In addition, the in vitro antioxidant capacity and the inhibition of acetylcholinesterase activity of NA-beer and its two main ingredients, silicon and hops, were evaluated [123].

Results of that study clearly show that the incorporation of aluminum nitrate plus NA-beer or its hops and silicon components significantly reduced the negative effects caused by aluminum nitrate administration on the behavior of rats and the brain’s inflammatory and antioxidant markers. The behavior assessment was performed according to a standard battery test. In the hole–board task, we evaluated curiosity, immobility time, grooming frequency, and the defecation index. Grooming was interpreted as a way to release tension, defecation rate indicated the emotive grade as an intestinal tonus and peristalsis increase, and immobility was related to transitory hyperactivity [167]. The pain threshold was evaluated throughout the hot plate test [168]. Merino et al. [123], in order to highlight differences between groups, performed MANOVA tests on the behavioral experiment battery tests and found that aluminum + NA-beer and aluminum + silicon did not significantly differ when compared to the control group, while aluminum + hops significantly differs in comparison with all other groups.

These tendencies were clearly demonstrated after testing behavior results by the principal components analysis for categorical data (CATPCA) in order to identify patterns and highlight relationships and to observe group distribution differences (optimum group scaling was: 1, control group; 2, aluminum nitrate-treated group; 3, aluminum nitrate plus non-alcoholic beer; 4, aluminum nitrate plus hops extract; 5, aluminum nitrate plus silicon). 

**Figure 7:**
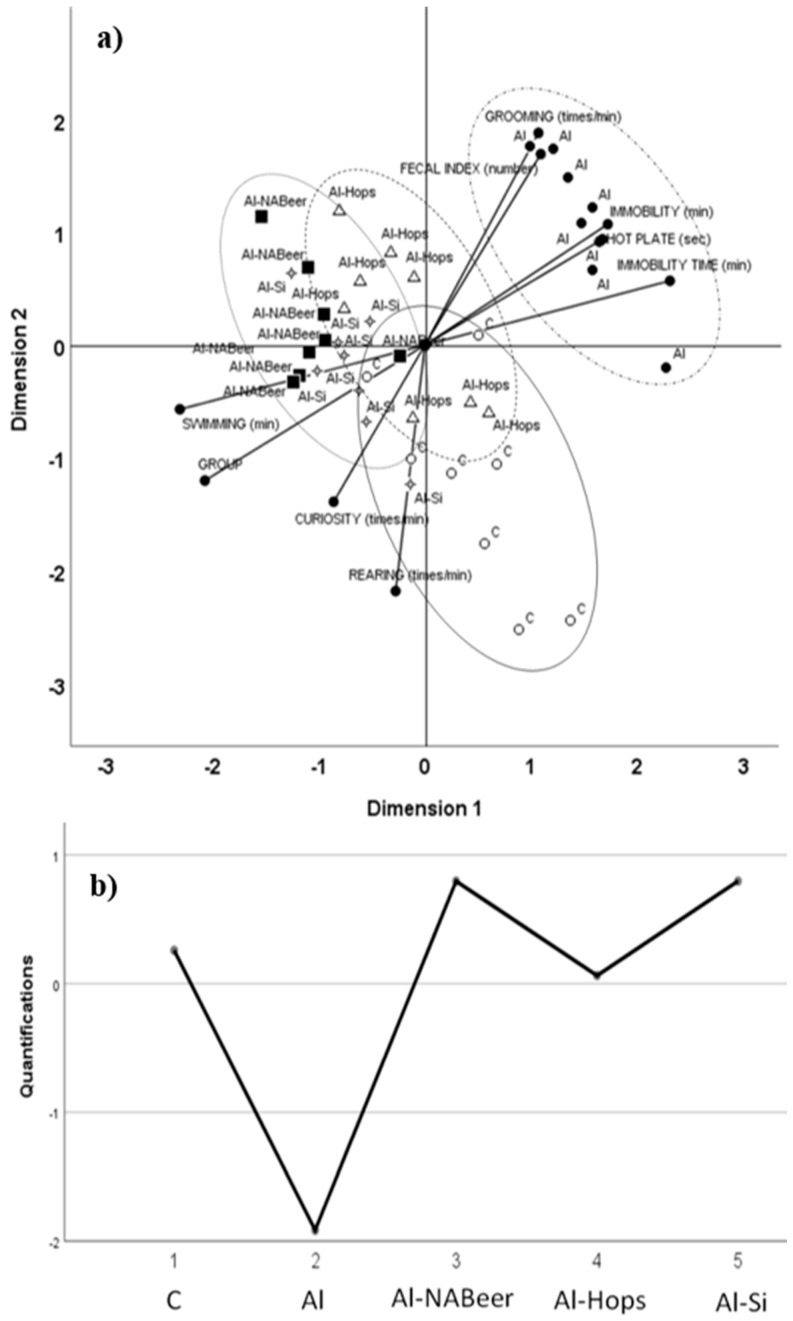
(**a**) Categorical principal component analysis (CATPCA) scatter biplot using groups and nine behavioral variables: rearing, immobility, curiosity, grooming, fecal index, swimming time at the forced swimming test (swimming), immobility time at the forced swimming test, and reaction time (hot plate). (**b**) Optimal scaling level for the different groups: 1, control group; 2, aluminum-treated group; 3, aluminum plus non-alcoholic beer; 4, aluminum plus hops extract; and 5, aluminum plus silicon. The first and second dimensions accounted for 43.2% and 31.1% of variance, respectively (3.89 and 2.80, respective Eigen values). The ellipses drown surround data for each of the different experimental groups [123].

On the basis of a stability study, two major components were found that explained 73.3% of total data variance (43.2% for the first one and 31.1% for the second one (Figure 7). Two subscales, positive and negative, for each component, explaining the contribution to the model, were drawn. For the first dimension, forced swimming and group scaling were the variables that most negatively contributed, while immobility time at forced swimming, immobility, and reaction time at the hot plate test, the variables that most positively contributed to the model. For the second dimension, rearing, curiosity, and group scaling most negatively contributed, while grooming, fecal index, and immobility most positively contributed to the model. The ellipses drawn help to identify data for the different experimental groups in comparison to their control counterpart and aluminum groups. The first dimension separates aluminum behavioral data from those of aluminum + silicon and aluminum + NA-beer. The second dimension separates the behavioral data of the aluminum group from those of the control group. Results also suggest a new role for NA-beer and its components, as the administration of NA-beer, hops, and silicon together with aluminum nitrate was effective in preventing the detrimental effect of this metal on memory decline. This preventive effect was also clearly shown in the CATPCA test evaluating behavior as the aluminum group data were sharply separated from the control and all treated groups. In addition, some related measurements (e.g., hot plate time reaction and fecal index changes) contributed similarly to the multivariate model applied, supporting the validity of the battery test performed in the present study.

According to relevant research, aluminum interacts with the cholinergic system, acting as a cholinotoxin. The intensification of inflammation and the interference with cholinergic projection functions may represent the way by which it contributes to pathological processes in Alzheimer’s disease, leading to learning and memory deficits [169] and explaining the negative effects on curiosity, immobility time, grooming frequency, defecation rate, and forced swimming on aluminum intoxicated rats observed in our study.

The in vivo test showed that behavioral improvements observed after the administration of NA-beer and its components in this study appear to be clearly associated with the in vitro results obtained from AChE and buthylcholinesterase (BChE) by our research group testing silicon in human neuroblastoma cells [128], suggesting that potential improvements in cholinesterase levels were involved and that silicon was one of the major factors responsible for this inhibition effect. In fact, Jacqmin-Gadda et al. [170] indicated that an association exists between cognitive impairment and brain pH, aluminum, and silica. Noremberg et al. [18] described a protective effect of silicon on the hippocampus (region of the brain traditionally linked with learning and memory control) and cerebellum against cellular damage caused by aluminum-induced oxidative stress, by the measure of lipoperoxides (LPO). An increase of AChE activity was observed in the aluminum-treated group in the cerebellum, whereas a decrease of this enzyme activity was observed in the cortex and hippocampus in the aluminum and aluminum + silicon groups.

In line with the González-Muñoz et al. [43] study, aluminum nitrate intoxication impairs the brain antioxidant status. Noremberg et al. [18], based on many investigations, suggested that the brain may be particularly vulnerable to oxidative damage due the relationship between aluminum accumulation and oxidative damage in the brain [171]. As previously commented on, although aluminum is not a transition metal and therefore cannot initiate peroxidation, aluminum induces alteration in brain metal homeostasis [17], mainly affecting minerals with antioxidant properties [40]. High TBARS values and alteration in the antioxidant enzyme activity and expression observed in the brains of the aluminum group corroborate the pro-oxidant effects of aluminum and suggest the relative failure of antioxidant mechanisms. Martínez et al. [53], after a subchronic study, concluded that aluminum increased hippocampal reactive oxygen species and lipid peroxidation, reduced antioxidant capacity, and decreased AChE activity. This would explain the memory impairment and neurotoxicity.

**Figure 8:**
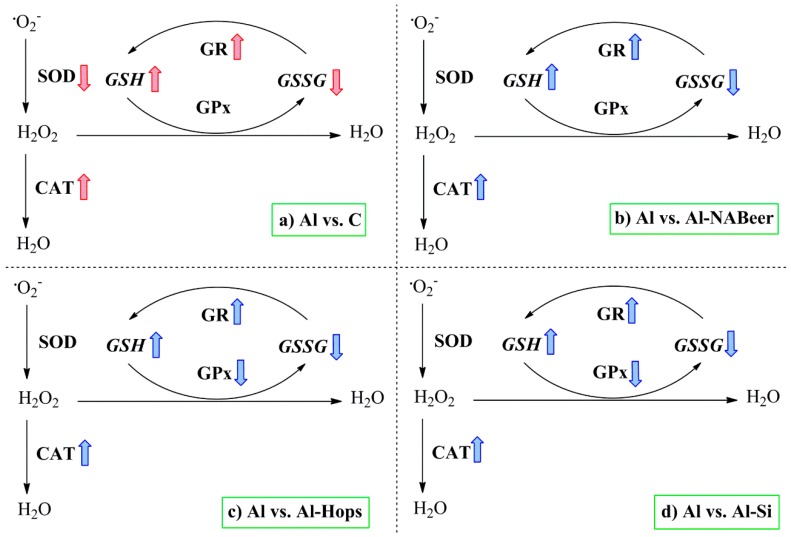
Summary of the main effect of aluminum intoxication on the antioxidant enzymes’ activities and expressions in the rats’ brains [123]. Colored arrows indicate significant differences caused by the aluminum-treated group when compared to the experimental groups. CAT, catalase; SOD, superoxide dismutase; GR, glutathione reductase; GPx, glutathione peroxidase; GSH, reduced glutathione; GSSG, oxidized glutathione. Subsections: (**a**) comparison between the Aluminium intoxicated group with their control counterparts; (**b**) comparison between the Aluminium intoxicated group with the Aluminium + Non-alcoholic beer group; (**c**) comparison between the Aluminium intoxicated group with the Aluminium + Hops extract group; (**d**) comparison between the Aluminium intoxicated group with the Aluminium + Silicon group.

Inhibition of TBARS by administration of aluminum + silicon or aluminum + NA-beer strongly suggests the neuroprotective properties of silicon and beer compounds against aluminum intoxication. According to Noremberg et al. [18], silicon may be considered an important protector against lipid peroxidation induced by Al^3+^. Figure 8 summarizes changes in the redox defense mechanism and the antioxidant enzymes’ activities and expressions. Aluminum intoxication significantly increased the SOD expression as a way to eliminate the produced superoxide ion, while due to the lower GR activity and expression and the higher GPx activity, GSH decreased. The conjoint administration of aluminum NA-beer, silicon, and hops displays similar profiles for antioxidant enzyme activity and expressions. NA-beer, hops extract, and silicon treatment appear to prevent aluminum-induced augmented lipid peroxidation and changes in GSH, GSSG, and redox index levels [123], probably by contributing both with antioxidants (e.g., phenolic compounds, flavonoids, tannins) (Table 2 and Table 3) or through the effect of silicon modulating the antioxidant enzyme expressions [43,150]. In addition, the conjoint administration of aluminum with hops, silicon or beer affected the inflammatory status in a quite similar manner. The differences observed between the battery test for behavior and antioxidant and inflammatory status clearly indicate the complexity of the effects tested and suggest the interaction of treatments on neurotransmitter changes (e.g., acetylcholine). Thus, hops clearly differentiate from beer and silicon at the behavior test with no differences with respect to the aluminum group, while exerting similar antioxidant and anti-inflammatory actions as silicon and Na-beer.

## 9. Conclusions

Taking into account all previous discussed results, we can reach the following conclusions:

- In vitro and in vivo models are plausible tools to study brain mechanisms related to changes in behavior, Alzheimer’s disease, and dementia.
- Aluminum induces several mechanisms engaged to brain damage and behavioral disturbances, through mechanisms that mainly involve apoptosis, tau phosphorylation, Aβ accumulation, ROS formation, necrosis of neuronal cells, regulation of metal imbalance, and changes in the antioxidant defense system.
- The conjoint addition of aluminum and beer, or its ingredients and compounds, proved that they can partially block the negative effects of neurodegeneration or neurotoxics, such as aluminum, in several cell, rodent, and human models.
- Due to its alcohol content, regular beer consumption can be non-adequate for some risk-group populations (pregnancy, children, people affected by liver diseases), and the consumption of non-alcoholic beer is highly recommended instead of regular beers.
- Given the results observed by our groups and others, dementia or the pathognomic factors can be blocked by promoting increased levels of silicon consumption in aluminum-intoxicated patients.
- As silicon is “attracted by” the presence of aluminum at the intestine and brain, among other body places, strategies should be formulated to adjust silicon consumption to aluminum exposure and to increase the brain uptake of silicon.

In summary, regular beer consumption could constitute a non-invasive preventive measure for the prevention of Alzheimer’s disease and other neurodegenerative diseases, since it is effective in reducing the aluminum body load, as well as in alleviating the mineral homeostasis imbalance in the brain and the pro-oxidant and pro-inflammatory effects induced by that metal. However, regular beer, due to its alcohol content, might not be adequate for consumption in all human beings. Thus, the study of non-alcoholic beer properties and their application as a preventive tool for degenerative disease is highly demanded.

## 10. Future Remarks

Future studies should be focused on analyzing how the presence of different levels of silicon, hops, polyphenols, melatonin in beer, and their interactions affect the metabolizing capability of both alcohol-metabolizing and antioxidant enzymes. In addition, the bioavailability of silicon differs depending on its food origin and chemical state [25].

Taking into account the ample beer consumption in the world and the increased prevalence of dementia, future investigation should be addressed at understanding the intimate mechanisms implicated in different alteration pathways leading to dementia and the most powerful mechanisms by which beer could slow down the development of dementia and Alzheimer’s disease.

Early markers (BDNF, pancreatic amyloid, Nrf2, receptor of advanced glycosylation end products—RAGE) should be tested to improve prevention and delete or avoid the negative consequences of dementia in animal models and even in human beings with a special study of the interaction of Aβ with glial cells. The role of beer and its compounds on these earlier markers should be investigated. The most beneficial compounds of beer should be concentrated and their bioavailability improved while the theoretical negative compounds deleted to obtain a functional beer capable of slowing down the dementia progression in animal models and human populations. 

Nutrigenomic studies should be performed and their results tested and studied in ample gen spectrum microchips. Genes related with carbohydrate and lipid metabolisms and adipogenesis (e.g., PPARγ), mitochondrogenesis, and autophagy will be researched. As some dietary compounds and lifestyle are implicated in the expression of BDNF [172], the role of beer and their compounds in this neurotrophic factor will also be studied. As ethanol differently affects people depending on its metabolization rate and that of acethaldehyde [173], studies on the possible deleterious/protective effect of regular beer consumption should address quick and slow alcohol metabolizers, studying the presence of gene polymorphisms for the alcohol dehydrogenase (ADH1B exon 3), aldehyde dehydrogenase (ALDH2 1), CAT, and for the major isoenzyme of the P450 isoforms (CYP2E1) and their interaction with major gene polymorphisms related to Alzheimer’s disease (e.g., ApoE, APP, PS-1, PS2, CLU, gen receptor of efrina A1 EPHA1, ATP Binding Cassette A7) [174]. In addition, differences in response to beer and beer compounds will be tested according to GWAS and EGWAS [175].

As recent studies have suggested a potential link between intestinal microflora composition and function and brain health [176,177,178], specific studies should also study how different types of beer can affect brain health through modifying colonic microflora profile and abundance.

The benefits of one-a-day alcoholic beer, low alcohol content, and non-alcoholic beer versus abstention in an ample range of ages both in male and female health or differently affected by different dementia degrees or by degenerative diseases having Alzheimer’s disease or dementia as comorbidity (e.g., diabetes) should be tested in long-term studies. 

## Figures and Tables

**Table 1:** Beer consumption in different countries.

Country	L Per Capita	Total National Consumption
China	29	43266
United States	74.8	24245
Brazil	60.4	12654
Germany	104.2	8412
Russia	58.60	8405
Mexico	65.1	7988
Japan	41.4	5251
Spain	84.8	3909
Poland	100.8	3892
Canada	57.7	2093
Argentina	49	1980
Czech Republic	143.3	1959
Netherlands	69.8	1186
Austria	106	928
Belgium	67.4	769

Per capita beer consumption by country of last data report by Kirin Holding Company at 2017 [79].

**Table 2:** Mean nutrient composition of regular beer.

Nutrient	Units	Mean Content/100 g
***Proximates***		
Energy	Kcal	91.97
Water	g	43
Protein	g	0.46
Total lipid (fat)	g	0.0
Carbohydrate, by difference	g	3.55
Fiber, total dietary	g	0.0
***Minerals***		
Calcium (Ca)	mg	4
Cooper (Cu)	mg	0.01
Iron (Fe)	mg	0.02
Magnesium (Mg)	mg	6
Manganese (Mn)	mg	0.02
Silicon (Si)	mg	1.92
Selenium (Se)	µg	0.6
Fluoride (F)	µg	44.2
Phosphorus (P)	mg	14
Potassium (K)	mg	27
Sodium (Na)	mg	4
Zinc (Zn)	mg	0.01
***Vitamins***		
Vitamin C, total ascorbic acid	mg	0.0
Thiamin B1	mg	0.005
Riboflavin B2	mg	0.025
Niacin B3	mg	0.513
Pantothenic acid B5	mg	0.041
Vitamin B-6	mg	0.046
Folate, DFE	µg	6
Choline, total	mg	10.1
Cobalamine B12	µg	0.0
Vitamin A, RAE	µg	0.0
Vitamin E (α-tocophenol)	mg	0.0
Vitamin D	IU	0.0
Vitamin K (phylloquinine)	µg	0.0
***Lipids***		
Saturated fatty acids	g	0
Monounsaturated fatty acids	g	0
Polyunsaturated fatty acids	g	0
Cholesterol	mg	0
***Aminoacids***		
Alanine	g	0.012
Aspartic acid	g	0.016
Glutamic acid	g	0.047
Glycine	g	0.013
Proline	g	0.035
***Ethyl alcohol***	g	3.9 *

Mean content of each component/100 g beer. Proximate values include ales, lagers, porters, premium beers, and stouts. Other nutrients based on lager samples. Data from the National Nutrient Database for Standard Reference (USDA) [107]. * May vary according to the type of beer.

**Table 3:** Phenolic compounds in beer

Phenolic Compound (g/100 mL)			
***Alkylmethoxyphenols***		5-Caffeoylquinic acid	0.08
4-Vinylguaiachol	0.15	Caffeic acid	0.03
***Alkylphenols***		Ferulic acid	0.26
3-Methylcatechol	1.00 × 10^−4^	Sinapic acid	0.02
4-Ethylcatechol	6.00 × 10^−4^	*Chalcones*	
4-Vinyphenol	4.53 × 10^−3^	Xanthohumol	1.41 × 10^−3^
***Hydroxybenzaldehydes***		*Flavanones*	
Vanillin	0.02	Isoxanthohumol	0.04
***Hydroxybenzoketones***		Naringin	7.5 × 10^−4^
2,3-Dihydroxy-1-guaiacylpropanone	3.4 × 10^−3^	8-Prenylnaringenin	1.04 × 10^−3^
***Hidroxycoumarins***		6-Prenylnaringenin	2.59 × 10^−3^
Esculin	0.02	6-Genarnylnaringenin	4.29 × 10^−4^
Umbelliferone	1.67 × 10^−3^	*Hydroxyphenylacetic acids*	
4-Hidroxycoumarin	0.11	4-Hydroxyphenilacetic acid	0.03
***Hidroxyphenylacetic acids***		Homovanillic acid	0.05
4-Hydroxyphenylacetic acid	0.03	Tyrosol	0.32
Homovanillic acid	0.05	(+)-Catechin	0.11
***Benzoic acid derivatives***		(-)-Epicatechin	0.06
Gallic acid 3-O-gallate	0.3	Procyanidin dimer B3	0.16
2,6-Dihydroxybenzoic acid	0.09	Prodelphinidin trimer GCGCC	0.04
2-Hydroxybenzoic acid	0.20	Prodelphinidin trimer GCCC	1.00 × 10^−2^
3-Hydroxybenzoic acid	0.03	Prodelphinidin trimer CGCC	0.02
3,5-Dihydroxybenzoic acid	0.03	*Flavonols*	
Syringic acid	0.02	Quercetin (3-O-arabinoside)	5.83 × 10^−4^
Protocatechuic acid	0.05	Quercetin	6.67 × 10^−3^
Vanillic acid	0.07	3,7-Dimetilquercitin	2.50 × 10^−4^
Gallic acid	0.07	Myricetin	6.67 × 10^−4^
Gentisic acid	0.03	Quercetin (3-O-rutinoside)	0.09
***Cinnamic acids***		*Isoflavonoids*	
*p*-Coumaric acid	0.10	Daidzein	0.005
*m*-Coumaric acid	0.02	Genistein	0.01
*o*- Coumaric acid	0.15	Biochanin A	0.005
4-Caffeoylquinic acid	0.01	*Flavones*	
***Other polyphenols***		Apigenin	4.17 × 10^−3^
Catechol	1.10 × 10^−3^	α-acids (humolones)	0.17
Pyrogallol	4.70 × 10^−3^	Iso-α-acids (iso-humolones)	0.06 × 10^−10^

Mean value in g/100 mL of beer. Modified from Arranz et al. [88].

**Table 4:** Main in vivo and in vitro studies of beer and its compounds’ activity on neurodegenerative diseases.

Compound	Species and Trial Mode	Formulation and Doses	Results	Reference
Beer	Clinical trial in humans with 125 males of the Helsinki Sudden Death autopsy	Total life consumption	Neuroprotective effect by a possible depletion of Aβ aggregation in brain	Kok et al. [111]
Beer	Cohort study with 360 patients in early AD, biannually evaluation up to 19–28 years.	Heavy drinkers (≥8 alcoholic drinks/week); mild-moderate drinkers (1–7 alcoholic drinks/week); abstainers	Increasing standard drinks of hard liquor, but not beer or wine, was associated with a faster rate of cognitive decline, such as AD	Heymann et al [114]
Beer	Cardiovascular Health Study, cohort study with 5888 men and women aged ≥65 years and 5–7 years follow up.	12oz of beer with later magnetic resonance imaging of the brain and cognitive capacity evaluation.	Limited beer consumption resulted in a decreased risk of dementia or AD	Mukamal et al. [115]
Beer	Cohort study with 980 community-dwelling individuals aged ≥65 years without dementia at baseline, annually evaluation.	Light drinkers (1 serving/month to 6 servings/week); moderate drinkers (1–3 servings/day); heavy drinkers (≥4 servings/day)	Light to moderate alcohol intake was associated with a lower risk of dementia and AD, whereas intake of beer and liquor was not associated with incident dementia.	Luchsinger et al. [116]
Beer	Review of the observational studies, trials, reviews, and meta-analyses in humans	Review from 45 reports since the early 1990’s	More than half of the papers indicate that low consumption of beer reduced the risk of dementia. While a minority suggests the risk of neurodegeneration due to its ethanol content	Collins et al. [112]
Beer	Immune response evaluation in human peripheral blood mononuclear cells with 48-h treatment	Different beer types with 2%–4% (*v*/*v*) ethanol	Beer reduces the production of neopterin and the tryptophan degradation. Its immunosuppressive capacity seems related to its anti-inflammatory mechanisms.	Winkler et al. [117]
Beer	In vitro experiments of pure samples	Electron paramagnetic resonance spectroscopy and antioxidant activity of different types of beer	Beers exhibit antioxidant properties	Polak et al. [118]
Alcohol	Review of the human, rodents and cell culture neuroprotection evaluations and epidemiological studies	Wine, beer, and liquor administration and consumption	Alcohol-dependent neuroprotected state appears linked to an activation of signal transduction processes of reactive oxygen species. The alcohol intake ameliorates inflammatory pathways and increases hippocampal acetylcholine release. Alcohol exposure is inversely associated with dementia through protective changes in cerebral vasculature	Collins et al. [112]
Beer/silicon	Human intake with 6-h bioavailability evaluation of silicon-enriched beer	0.6 L beer containing 22.5 mg Si and 4.6% (*v*/*v*) ethanol	Silicon in beer in monomeric form, is readily bioavailable in healthy volunteers	Sripanyakorn et al. [119]
Beer/silicic acid	Acute three-day study with male NMRI mice	Equivalent to moderate-high consumption in humans (1 L/day; 55 g alcohol/day).	Beer, mainly associated with its silicon content, reduces dietary aluminum toxicokinetics and bioavailability through a reduction of aluminum uptake in the digestive tract and by increasing its fecal excretion	Peña et al. [120]
Beer/silicon	Male NMRI mice on 3-month trial with neuroprotective evaluation	2.5 mL beer/per week (5.5% (*v*/*v*)), and 40 mg silicon/L/day	Silicon appears to be effective in preventing aluminum accumulation in mouse’s brain. Nonetheless, silicon could act either as neuroprotector or neurotoxic	Granero et al. [121]
Beer/silicic acid	Male NMRI mice on 3-month evaluation	450 mg of aluminum nitrate,0.5 mL beer* (5.5% (*v*/*v*))/day, and 9 µg silicon/day *equivalent to moderate to high consumption in humans (1 L/day)	Beer consumption, and its content on bioavailable silicon, reduces the accumulation of aluminum in the body and brain tissue, the lipid peroxidation, and protected against the neurotoxic effects through the regulation of antioxidant enzymes	González-Muñoz et al. [48,122]
Beer/silicic acid	Male NMRI mice on 3-month trial	450 mg of aluminum nitrate,0.5 mL beer* (5.5% (*v*/*v*))/day, and 9 µg silicon/day *equivalent to moderate to high consumption in humans (1 L/day)	Silicic acid and beer block the metal imbalance, inflammation, and antioxidant defense impairment induced by aluminum intoxication in the brain	González-Muñoz et al. [43]
Non-alcoholic beer/hops extract/organic silicon (Silicium organique G57™)	Male Wistar rats on 3-month trial with behavioral, organs, and in vitro studies of neurodegeneration	450 µg aluminum nitrate/kg/day; 2 mg hops extract/day; 250 µg silicon/day; 5 mL NA-beer/day* *equivalent to moderate/high consumption in humans (1 L/day)	NA-beer, hops, and silicon ameliorated behavioral modifications, blocked the negative effect on the in vivo and in vitro antioxidant status, and reduced the inflammation markers in brain induced by aluminum intoxication	Merino et al. [123]
Hops extract	Homozygous transgenic mice (V717F) and heterozygous transgenic mice (V717F/P267S) on a 2-, 6-, 11-, and 18-month trial, and HEK293A cell culture	Hops extract added to drinking water at a dose of 2 g extract/L	Hops extract reduced Alzheimer’s phenotypes in mice and prevented the emotional disturbance at the 18 months AD-mice. The extract significantly reduced Aβ production in cultured cells and presented γ-secretase inhibitory activity	Sasaoka et al. [124]
Hops pellets	Hops pellets to obtain its chemical characterization	Chemical and quantitative determination of hops compounds	The presence of resveratrol in hops highlights the potential health-promoting effect of moderate beer consumption	Callemien et al. [125]
Silicon	Human trial in 7598 women ≥75 years for evaluation of cognitive function and neuroprotective effect	Silica content in drinking water	Silicon in drinking water might reduce the risk of AD	Gillette-Guyonnet et al. [126]
Silicon	Review from human trials that evaluate the neuroprotective effect of silica in drinking water	Reviews from tidies of silica in drinking water	Aluminum in water seems to have a deleterious effect when the silica concentrations were low, while the risk of AD was reduced in subjects who had higher daily silica intake	Gillette-Guyonnet et al. [127]
Silicon	Male Wistar rats on a 12-week trial	0.5 mg aluminum/kg/day, and 2 mg silicon/kg/day	Silicon is considered an important protector against lipid peroxidation induced by aluminum intake	Noremberg et al. [18]
Silicon	SH-5HSY neuroblastoma cells line with a 24-h treatment	Ladder concentration of 50–250 ng silicon/mL	Silicon treatment reduced TBARS levels, it also may act as neuroprotector by inducing antiapoptotic effects at low doses and may act as neurotoxic by regulating necrosis and apoptosis mechanisms at high doses.	Garcimartín et al. [128]
Beer/melatonin	Human trial with healthy volunteers (4 men and 3 women) aged 20 to 30 years.	Different beer brands with diverse ethanol content	Melatonin contained in beer showed antioxidant, oncostatic, and immune enhancer activities	Maldonado et al. [129]
Melatonin	Review of the main effects on AD pathology	Main research publications on the melatonin pathological mechanisms related to AD through different approaches	Prevention of amyloid overproduction, reduction of tau phosphorylation, antioxidant ability, modulates inflammation, anticholinesterase agent, prevents mitochondrial damage and apoptosis.	Rosales-Corral et al. [130]
Melatonin	Transgenic mice Tg2576 on an 8-, 9.5-, 11-, and 15.5-month survival study trial to evaluate neuroprotective effect	0.5 mg/mL melatonin administration to obtain Aβ measurements in brain, quantitative immunoblots of APP levels, and nitrotyrosine measurements	The melatonin administration proved a reduction of major AD markers and brain Aβ levels	Matsubara et al. [131]
Melatonin	Male swiss albino mice in a 5-day neuroprotective effect evaluation experiment	50 mg melatonin/kg body weight and 5µg Aβ_42-1_ intracerebroventricularly administrated	The treatment reduced Aβ-induced oxidative stress, related to ROS and proinflammatory cytokines IL6 and IL1-β, and the intracellular calcium levels and acetylcholinesterase activity in the neocortex and hippocampus regions	Masilamoni et al. [132]
Melatonin	Review of the in vivo and in vitro studies	Reviews about the effects on the prevention of neurodegenerative diseases and their molecular mechanism	Melatonin is significantly decreased in elderly AD individuals and associated with the emergence of AD, it exhibits a protective effect on the cholinergic system and protects brain neurons from damage and death by increasing viability in hippocampal neurons and glial cells	Hornedo-Ortega et al. [133]
Phenolic compounds from beer	In vitro anti-AChE and anti-BChE activities of simple phenolic acids	Phenolic solutions at their beer concentration. IC50 values at 336 and 160 mM calculated for AChE and BChE, respectively	Phenolic acids from beer can play a role in neuroprotection by through an inhibition of cholinesterases	Szwajgier [134]
Phenolic compounds from malt	In vitro studies of the phenolic fraction profile from several malt types.	Phenolic solution at different concentrations to inhibit AChE and BChE enzymes (~0.38–1 mM/L)	The main phenolic compounds from malt (ferulic acid, p-coumaric, 4-hydroxybenzoic, and sinapic acids). Among them, the ferulic and *p*-coumaric acids showed a high neuroprotective role and can be considered as possible anti-AD agents	Szwajgier and Borowiec [135]
Phenolic compounds from beer	Female Tg2576 mice on a 14 months trial to evaluate the pathology of AD	Different diets including 0.5% phenolic compounds evaluated through immunohistochemistry and morphometry of Aβ deposits	The extracted phenolic compounds prevent AD pathology development through the regulation of Aβ aggregation pathway	Hamaguchi et al. [136]
Xanthohumol	Wild-type murine neuroblastoma Neuro2a cells (N2a/WT) and N2a stably transfected with human APP Swedish mutant (N2a/APP) on a 24-h treatment	0–25 μM Xanthohumol in cell culture and later comparative proteomics, immunocytochemistry of Aβ_1-40_ and Aβ_1-42_	Xanthohumol suppresses Aβ production and tau hyperphosphorylation via APP processing and the GSK-3β pathway. Thus, it may have potential effects for the treatment of AD	Huang et al. [137]
Iso-α-acids	Alzheimer’s model in 5xFAD mice on a three-month period to evaluate cognitive function in the progression of dementia	0.05% (*w*/*w*) of the iso-α-acids	The iso-α-acids suppressed the neuroinflammation markers IL-1β and chemokine, and improve cognitive function	Ano et al. [138]
Iso-α-acids	Male Crl:CD1(ICR) mice, vagotomized male ICR mice, and Sprague-Dawley (SD) rats on a 3-month period to evaluate cognitive function test, especially hippocampus-dependent memory	1 mg/kg iso-α-acids	Iso-α-acids activate dopamine D1 receptor-signaling in the hippocampus and improves spatial and object recognition memory functions	Ano et al. [139,140]
Iso-α-acids	Male C57BL/6J mice treated for 3 months	Dietary intake of 0.05% (*w*/*w*) iso-α-acids to evaluate episodic and spatial memory and microglia analysis	Reduced inflammation in the brain and prevent the cognitive impairment associated with normal aging	Ano et al. [140]
Iso-α-acids	Male C57BL/6J mice on an AD model (5xFAD transgenic) on a 7-day trial	1 mg/kg iso-α-acids and later transcriptome analysis	Reduced Aβ in the brain and increased the expression of transthyretin in the hippocampus, thus displayed protective effects AD pathologies	Fukuda et al. [141]
Beer/iso-α-acids from hops extract	Male C57BL/6J mice on a long-term cognitive evaluation trial	1 mg extract/kg equivalent to 4.8 mg/day in humans (60 kg body weight) or 0.17–0.3 L/day of beers	Iso-α-acids could improve working memory in dementia and visual/reversal discrimination learning, which are considered high-order cognitive functions.	Ayabe et al. [142]

AChE, acetyl cholinesterase; AD, Alzheimer’s disease; Aβ, amyloid-β; Al, aluminum; BChE, butylcholinesterase; ChAT, choline acetyltransferase; HDL, high density lipoproteins; ROS, reactive oxygen species; Si, silicon; TNFα, tumor necrosis factor alpha.

## Abbreviations

Aβ, amyloid beta peptide; AChE, acethylcholinesterase; APO-E, apolipoprotein E; AP, amyloid beta protein; APP, amyloid precursor protein; BChE, buthylcholinesterase; BDNF, brain-derived neurotrophic factor; CAT, catalase; GPx, glutathione peroxidase; GSH, reduced glutathione; GSSG, oxidized glutathione; LPO, lipoperoxides; MD, Mediterranean diet; NA-beer, non-alcoholic beer; PPAR-γ, peroxisome proliferator-activated receptor gamma; IFN-γ, pro-inflammatory cytokine interferon-gamma; ROS, reactive oxygen species; SOD, superoxide dismutase; TBARS, tiobarbituric acid reactive substances; TNFα, tumor necrosis factor-alpha; HSV-1, type I herpes simplex virus.
